# Pharmacogenetic testing to improve care in the emergency department

**DOI:** 10.3389/fphar.2026.1866821

**Published:** 2026-10-21

**Authors:** Hayley T. Gartner, Julio D. Duarte, Sophia Sheikh

**Affiliations:** 1 Department of Pharmacy, University of Florida Health, Jacksonville, FL, United States; 2 Center for Pharmacogenomics and Precision Medicine, University of Florida College of Pharmacy, Gainesville, FL, United States; 3 Department of Pharmacotherapy and Translational Research, University of Florida College of Pharmacy, Gainesville, FL, United States; 4 Department of Pharmacy Practice and Science, The Ohio State University College of Pharmacy, Columbus, OH, United States; 5 Department of Emergency Medicine, University of Florida College of Medicine-Jacksonville, Jacksonville, FL, United States

**Keywords:** clinical decision support, Drug–gene interactions, emergency medicine, pharmacogenetics, pharmacogenomics, precision medicine

## Abstract

Nearly 20% of emergency department (ED) encounters involve medications with actionable pharmacogenomic (PGx) guidance available, and population-based studies suggest over 90% of patients carry at least one PGx variant potentially affecting drug response. Despite this, PGx testing in emergency medicine remains rare. This perspective summarizes current evidence supporting PGx implementation in the ED, key barriers to integration, and future directions for PGx testing in acute care. Feasibility studies demonstrate that PGx testing can be incorporated into ED workflows. Observational data suggest clinically important associations between *CYP2C19* variability and clopidogrel-related bleeding, CYP2D6 metabolism and opioid-related ED visits, and a high prevalence of actionable gene–drug interactions in older adults presenting with falls. In pediatric psychiatric populations, combinatorial PGx testing has been associated with differences in drug exposure and clinical outcomes. However, no randomized controlled trials testing the clinical utility of PGx testing have been conducted in ED populations. Key barriers to implementation include prolonged PGx testing turnaround times, lack of standardized ordering workflows, limited clinician familiarity with PGx interpretation, inconsistent reimbursement, and challenges integrating results into clinical decision support systems. Future priorities should include prospective interventional trials assessing clinical outcomes, targeted implementation focused on high-risk gene-drug pairs, and incorporation of PGx education into emergency medicine training. Although current evidence is largely feasibility-based, the ED represents a potentially high-yield setting for PGx integration given frequent exposure to actionable medications and the high burden of potentially preventable adverse drug events.

## 1 Introduction

Adverse drug events (ADEs) represent a substantial and ongoing challenge to patient safety in the United States. Each year, more than 1.5 million individuals are treated in an emergency department (ED) for ADEs, and approximately 500,000 of these encounters result in hospitalization (Medication Safety Program, 2024). In addition, an estimated 2% of patients experience an ADE during the ED encounter itself (Hafner et al., 2002). ED visits attributable to ADEs are associated with increased healthcare costs, poorer clinical outcomes, and higher hospital admission rates, underscoring the significant burden that medication-related harm places on both patients and the healthcare system (Hohl et al., 2011; Queneau et al., 2007).

ADEs can include adverse drug reactions (ADRs), lack of medication efficacy, medication errors, drug interactions, and medication misuse. A meaningful proportion of ADEs are potentially preventable and arise from factors such as human error, inappropriate prescribing, and interindividual variability in drug metabolism and response (Hakkarainen et al., 2012). Genetic variation in pharmacogenes is an important contributor to this variability and can influence both drug efficacy and the risk for adverse reactions. These issues may be particularly relevant in the ED, an acute care environment in which medically complex and high-risk patients are frequently treated with medications that have clinically actionable pharmacogenomic (PGx) recommendations. Analysis of National Hospital Ambulatory Medical Care Survey (NHAMCS) data from 2010–2015 estimated that more than 148 million ED visits in the U.S. (nearly 20% of all ED visits during the study period) involved ordering medications with PGx guidance available (Limkakeng et al., 2021). These medications include ondansetron, proton pump inhibitors, and medications categorized as high-alert by the Institute for Safe Medication Practices (ISMP, 2026) such as opioids and warfarin (Limkakeng et al., 2021). Additionally, population-based studies indicate that greater than 90% of U.S. adults carry at least one pharmacogene variant associated with altered drug response, highlighting the widespread relevance of PGx in clinical care (Van Driest et al., 2014).

Although not all ADEs are genetically mediated, PGx testing has emerged as a promising strategy to reduce preventable medication-related harm and optimize therapeutic outcomes. By identifying clinically relevant genetic variants before medication exposure, PGx testing may enable clinicians to further individualize drug selection and dosing, thereby improving both safety and effectiveness. In recent years, interest has grown in the potential role of PGx testing within the ED, particularly as the ED increasingly functions as a critical access point to the healthcare system for several patient populations.

Despite the potential impact of PGx testing in the ED, evidence supporting its implementation in this setting remains limited. Nevertheless, the current limited evidence suggests that PGx testing can be integrated into ED workflows and may provide meaningful clinically relevant information. Importantly, implementing PGx testing may allow the ED to serve as a gateway for integrating genomic information into longitudinal patient care.

## 2 Evidence supporting pharmacogenomic testing in the ED

### 2.1 Feasibility of testing in the ED setting/population

PGx testing appears feasible across a wide spectrum of emergency department environments and among diverse patient groups who routinely receive medications with PGx relevance. Because the ED is a high-risk prescribing setting where rapid, accurate decisions are critical, preemptive PGx data has the potential to accelerate and improve the safety of time sensitive care. In the acute cardiovascular ED setting, point-of-care *CYP2C19* genotyping using rapid, buccal swab-based testing has proven feasible, enabling timely guidance of antiplatelet therapy selection in patients with acute coronary syndromes, with results available to inform early treatment decisions (Stimpfle et al., 2014). This study demonstrated that point-of-care *CYP2C19* genotyping can effectively function within real-world time constraints of acute care and directly inform antiplatelet selection in the ED.

Implementation of PGx testing may also be feasible in the pediatric ED setting. A recent study enrolled 100 children aged 3–18 years old presenting to the ED with acute psychiatric or behavioral crises who were prescribed psychotropic medications (Ghosh et al., 2023). The authors observed that 73% were taking at least one medication with a gene-drug interaction and concluded that combinatorial PGx testing might predict medication blood levels and associated clinical outcomes (Ghosh et al., 2023). Importantly, among patients who presented specifically for depression or suicidal ideations, those who were prescribed PGx concordant medications (without gene-drug interactions) experienced significantly better clinical improvement scores compared to those taking discordant medications as measured by parent-rated Clinical Global Impression assessments (Ghosh et al., 2023). The feasibility of integrating PGx testing into pediatric ED workflows has also been evaluated. A pediatric asthma feasibility study conducted in the ED found that both the integration of DNA collection *via* buccal swabs for PGx testing and spirometry into acute care workflows were well-accepted. Nearly 100% of physicians, 90% of nurses, and 90% of respiratory therapists reported that this new workflow had no significant negative impact on their clinical activities (Fishe et al., 2020). Although these results suggest that PGx testing in this vulnerable population is feasible, the studies’ small sample sizes and use of subjective outcome measures limit these findings and underscore the need for prospective validation in larger cohorts. For example, outcomes such as improved depression scores, reduced psychiatric medication changes, or reductions in healthcare usage (such as clinic visits or emergency care) would provide a stronger evidence base supporting the benefit of PGx testing in this population.

### 2.2 Predicting risk of adverse drug reactions

PGx testing has shown promise in predicting ADR risk in patients. The PREPARE study demonstrated that genotype-guided treatment using a 12-gene PGx panel significantly reduced clinically relevant ADRs by 30% in a large European cluster-randomized trial (Swen et al., 2023). However, this study was conducted across diverse healthcare settings including hospitals and community health centers, not specifically focus on ED patients. On the other hand, a large retrospective study of a general healthcare system population found no association between PGx-predicted drug response phenotypes and hospitalization or ED visits (Takahashi et al., 2021). However, this study did not target patients who previously visited an ED or possessed a specific disease, and it did not differentiate whether or not ED visits were ADR related. To date, there are limited studies investigating the relationship between PGx testing and its ability to reduce ED visits and/or recidivism; thus, this represents a potential new direction for future study.

The ADRED study analyzed 776 ADR cases presenting to EDs and reported that genotype-predicted CYP2C19 activity was significantly associated with bleeding events in patients taking clopidogrel (Just et al., 2020). In their analysis, higher CYP2C19 activity was associated with a nearly 5-fold increased odds of clopidogrel-related bleeding ADRs (Just et al., 2020). The study also identified a trend between phenprocoumon-risk profiles and related ADRs (Just et al., 2020). The investigators concluded that PGx impact on serious ADRs may be highest for drugs that cannot be easily monitored or those that do not provoke mild ADR symptoms quickly, suggesting that patients requiring medications with significant PGx variability, may particularly benefit from PGx testing (Just et al., 2020). While these findings support a potential role for PGx testing to predict ADRs in the ED, the study’s observational design and small drug scope are likely not sufficient to drive clinical implementation on their own. However, PGx-guided strategies could be reasonably prioritized in future studies, particularly for medications commonly used in the ED setting.

### 2.3 Prescribing of CYP2D6-dependent opioid medications

*CYP2D6* PGx has significant implications for prescribing opioid medications in the ED setting, as reduced CYP2D6 activity is associated with increased pain related ED visits among patients taking CYP2D6-metabolized opioids. A large retrospective cohort study found that patients taking hydrocodone, tramadol, or codeine who were concurrently exposed to antidepressants that were also CYP2D6-inhibitors had 75% higher odds of pain related ED visits compared to those on non-inhibiting antidepressants, with similar findings in patients taking oxycodone (Nahid et al., 2024). The same group subsequently conducted a national cohort study of over 31,000 patients from the All of Us Research Program and essentially confirmed these findings. They reported that patients who were CYP2D6 intermediate or poor metabolizers (predicted by both genotype and drug interactions) experienced almost 20% increassed risk of pain-related ED visits than those predicted to havenormal or increased CYP2D6 activity (Nahid et al., 2025). The investigators concluded that integrating *CYP2D6* genotype data and drug interaction considerations into clinical decision support tools could optimize pain management and potentially reduce opioid-related ED visits (Nahid et al., 2025). However, in a recent randomized pragmatic trial in post-operative acute pain patients, use of *CYP2D6* genotyping to inform opioid prescribing did not improve pain control or reduce opioid consumption compared to usual care (Cavallari et al., 2026). While not conducted in an ED setting, these recent results suggest that PGx may not provide significant benefit in all pain populations, particularly those using multi-modal pain management approaches. Emergency department pain management frequently incorporates multimodal analgesic strategies, and prescribing of concomitant medications could also affect a patient’s actual metabolic capacity at the time of prescribing. Phenoconversion--resulting from concomitant medications (such as CYP2D6 inhibitors), polypharmacy, or acute inflammatory states--can alter functional enzyme activity independent of genotype. Thus, genotype alone is insufficient to guide opioid selection in the emergency department and underscores the need for clinical decision support tools that incorporate concomitant medications and other patient-specific factors capable of modifying opioid response in real time.

### 2.4 Prescribing in older adults

PGx testing may hold particular relevance for older adults presenting to the ED, a population characterized by polypharmacy and heightened vulnerability to ADRs. A pilot study of 44 middle-aged and older adults discharged from the ED with opioid prescriptions for acute pain demonstrated the feasibility of recruiting socioeconomically disadvantaged and racially diverse older populations for PGx research in the ED setting; they found that pain interference may predict opioid-related falls or serious adverse effects within this population (Sheikh et al., 2023). More recently, a prospective study of 25 geriatric ED patients (median 81 years old) who sustained fall-related injuries found that 56% possessed potentially significant PGx gene-drug interactions, with 24% having one or more potentially serious interactions based on evidence-based guidelines and FDA-approved drug label information (Shih et al., 2025). Patients were prescribed a median of eight medications, with antihypertensives, statins, anticoagulants, and antiplatelet medications being the most common (Shih et al., 2025). While these findings suggest that older adult ED patients might particularly benefit from PGx testing, larger prospective studies are needed to determine whether PGx-guided prescribing in this setting improves clinical outcomes in this population.

### 2.5 Limitations of current literature

Current evidence supporting PGx testing in the ED is limited by significant methodological constraints, making it difficult to draw definitive conclusions about its clinical utility. Most critically, it is unknown if broad preemptive testing, that is not integrated with medication prescribing, meaningfully reduces ED utilization. Additionally, the lack of prospective randomized controlled trials specifically in the ED setting represents a major evidence gap. Most ED-based PGx studies to date have been observational feasibility studies or retrospective analyses rather than prospective studies or interventional clinical trials designed to demonstrate clinical utility and patient outcomes in the acute care setting. This limits the ability to make evidence-based decisions regarding implementation of PGx testing in emergency medicine practice.

## 3 Barriers preventing clinical implementation

In addition to a lack of studies assessing the potential clinical utility of deploying PGx testing in the ED, several other barriers exist that likely limit its implementation in this setting. These include financial, logistical, and knowledge barriers (Figure 1). Addressing these barriers is expected to facilitate expanded clinical implementation research in the ED.

**FIGURE 1:**
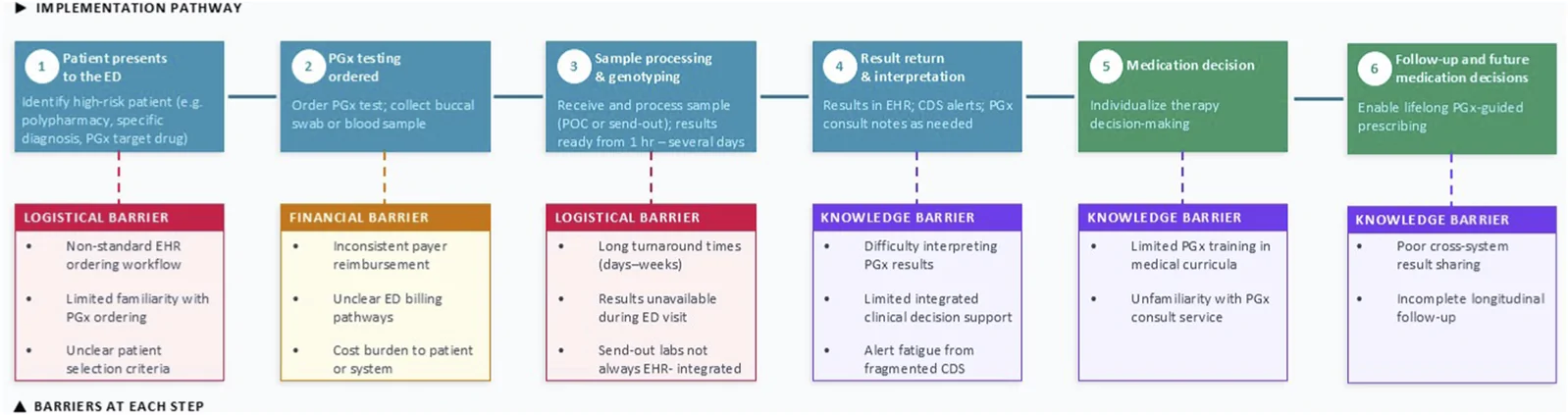
Suggested implementation pathway for implementing pharmacogenetics into emergency departments. Potential barriers are listed with each applicable implementation step.

### 3.1 Logistical barriers

Genetic testing is not routinely ordered in the ED and, in many institutions, its ordering and collection processes differ from those of standard laboratory tests. Genetic tests may be sent to laboratories not commonly used by the ED and thus have a different electronic health record (EHR) ordering process. For tests requiring buccal swab collection rather than blood, workflows for sample collection and submission may be less standardized in the ED. As a result, ED clinicians may be uncertain how to order PGx tests, and staff may be unfamiliar with proper buccal swab collection--a method not commonly used in ED practice. Many of these issues can be addressed by clinician and staff education and quick reference materials to help clinicians and support staff to retain the knowledge on how to order, collect, and submit samples for PGx testing. In-service presentations, online training videos, and one-on-one discussions can all be effective methods to disseminate this education. Additional support would likely be needed to assist with PGx test result interpretation (see *Knowledge Barriers* below).

In addition, the turnaround times for genetic tests are generally longer than standard biochemical laboratory tests–ranging from a few days to a week or more. Since patients generally do not stay in the ED for that long, it is unlikely that a patient would still be under the ED clinician’s care by the time the test results become available. This barrier might be amplified with the expansion of freestanding EDs that expand patient access but do not always possess the same on-site laboratory infrastructure that hospital EDs possess. Regardless of the ED setting, collaboration with the testing laboratory can often reduce turnaround times. However, current genetic testing technology often limits the ability to receive results fast enough for use in the ED. Because a majority of patients will not carry a genetic variant informing the specific drug being prescribed at that time, it might be harmful to delay prescribing to wait on the results of a PGx test for those patients. Thus, adapting the ED’s PGx use strategy might improve feasibility. One strategy would be to start PGx implementation with medications that can be informed by point-of-care testing, currently only available for *CYP2C19* (Genomadix CYP2C19 Genotyping, 2026). With an approximately 1-h turnaround time, results would likely be available before the patient leaves the ED. However, point-of-care testing requires additional workflows related to ordering, performing and interpreting tests that often differ from laboratory tests ordered from central institutional or commercial laboratories. There are also additional regulatory compliance requirements to be considered. Another strategy would be to collect a DNA sample while the patient is in the ED, but plan to use the results at their next follow-up visit. Because many ED patients follow up with a healthcare provider relatively soon after their ED visit, medication changes could be completed in a timely fashion for a large proportion of patients. Because genetic results are applicable for life, the results for patients who do not follow up could still be used at future healthcare provider visits.

Once available, PGx results should be acted upon, even if the patient has already been discharged from the ED. For patients who are admitted as inpatients within the same facility, this is a straightforward process. However, for patients who are transferred to another facility or discharged home from the ED, who bears the responsibility for acting on the PGx results may be less clear. However, EDs usually have systems in place to follow up on laboratory results (such as bacterial cultures) that become available after the patient is discharged, so these systems could be adapted to address this barrier. This could also be an opportunity for a PGx consult service to assist ED clinicians in overseeing this specific transition of care. The consult service could reach out to the treating clinician or even the patient if an applicable healthcare provider cannot be identified.

Because PGx results can be valid for the patient’s lifetime, mechanisms for making the results available long term across multiple healthcare systems are needed. While systems such as Epic’s Care Everywhere [https://www.epic.com/careeverywhere] allow participating systems using Epic EHR systems to view some test results, not all eligible health systems fully participate, and many others do not use the Epic EHR system. Thus, additional strategies are needed to more effectively disseminate PGx test results. One example is the use of a plastic card containing PGx results within a QR code that can be scanned by any clinician (Blagec et al., 2018). This could in the future evolve to cards stored on patients’ smartphones to reduce the risk of them being lost.

### 3.2 Financial barriers

Despite evidence suggesting that over 90% of patients harbor at least one PGx variant, there appears to be little appetite from third-party payers to reimburse for widespread preemptive PGx testing (Van Driest et al., 2014). Payers often cite the desire for more data showing cost-effectiveness and improved patient outcomes (which have not been required for most other laboratory tests). Efforts are underway to produce more evidence, as well as harmonize coverage policies across the U.S. (Empey et al., 2021). In addition, legislation mandating coverage for biomarker testing (such as PGx) at the state level are also developing (Lin et al., 2024). While these efforts progress, one strategy is to focus PGx implementation on patients most likely to benefit from testing in the near future. This could include targeting ED patients being prescribed medications with known PGx interactions, or those diagnosed with conditions commonly treated by such medications.

Another consideration is whether to order testing only for the gene(s) associated with the drug being considered. PGx testing panels that include multiple pharmacogenes cost only marginally more than single-gene tests, while providing results for several additional genes that might inform future prescribing decisions (Duarte et al., 2021). This often makes panel tests more cost efficient than single gene tests.

### 3.3 Knowledge barriers

Once PGx results become available, clinicians often report feeling uncomfortable interpreting the results and explaining them to patients (Mowbray et al., 2022). Interpretation of PGx results relies on standardized nomenclature and evidence-based translation frameworks. Organizations such as the Clinical Pharmacogenetics Implementation Consortium (CPIC; https://www.clinpgx.org/cpic) provide expected patient phenotypes based on genotype test results. For example, genes encoding drug metabolizing enzymes are categorized into predicted metabolizing phenotypes such as poor, intermediate, normal, rapid, and ultrarapid metabolizer. CPIC also provides recommendations on how to use these genotype-predicted phenotypes to inform relevant medication prescribing, enabling consistent interpretation across healthcare settings. They also provide standardized clinical decision support (CDS) language to use. However, the reported knowledge deficit likely decreases test ordering rates and adversely affects PGx adoption–particularly in specialties that do not often order genetic testing, such as emergency medicine. Several strategies have been undertaken to address this barrier. One is providing separate PGx expertise within the health system, often as a PGx consult service. These are usually led by clinical pharmacists and can be involved for pre-test and/or post-test consultations and PGx-based prescribing recommendations (Duarte et al., 2021). These PGx experts can also provide clinician education through online education modules or in-person training sessions.

Another (sometimes complementary) strategy is to develop EHR-based CDS to provide recommendations as clinicians order medications in real time. These can be developed using evidence-based guidelines and suggested CDS language from CPIC. The CDS ideally would provide concise recommendations and easily link the prescriber to more detailed source material if they want more information. Incorporation of these standardized frameworks into clinical decision support tools may help facilitate accurate and actionable PGx implementation, particularly in time-sensitive acute care environments.

## 4 Future directions

As genetic testing costs decrease and the evidence base grows, PGx testing is expected to become more prevalent in clinical practice. While this is primarily completed today with genotyping panels, it will likely shift to a genomic sequencing approach in the future. Whole exome sequencing is already being used clinically for rare disease diagnosis and for molecular diagnostics in cancer. A drawback to exome sequencing is its difficulty resolving gene duplications or pseudogenes, which can occur with *CYP2D6*. However, the ability to capture most clinically significant variation within a single test is the most efficient way to implement genetic testing; thus, the future of genetic testing will likely involve a single genomic/exomic sequencing test completed once in a patient’s lifetime and repeatedly queried for information based on their care at a particular moment. For instance, if a prescription is being considered, the patient’s exome can be queried to assess if there are any potential PGx interactions with that medication. Having these data available preemptively would be especially beneficial in the ED, where clinical decisions and medication orders are time sensitive.

To support a future where massive amounts of genomic data are available for each patient, more extensive and complex CDS systems must be developed. These should be able to consider several genetic variants that might affect the patient’s phenotype in different, potentially conflicting ways. Clear explanation of the evidence associated with each variant will be needed to help clinicians make informed decisions. However, genetics is just one piece of the clinical picture–more advanced CDS will be needed to help clinicians combine multiple types of clinical data. For instance, current CDS is available for drug-gene interactions (PGx) and separately for drug-drug interactions. A CDS system capable of combining these factors to provide a single recommendation could provide ED clinicians more useful information and simultaneously reduce alert fatigue.

In conclusion, ED patients appear to be a population that could particularly benefit from PGx testing. However, before widespread clinical implementation can be undertaken, additional research assessing clinical outcomes is needed to provide further evidence to support this hypothesis. Such data would also prepare ED clinicians for the future expansion of genomic medicine.

## Data Availability

The original contributions presented in the study are included in the article/supplementary material, further inquiries can be directed to the corresponding authors.
